# Finite element analysis of intramedullary nailing and double locking plate for treating extra-articular proximal tibial fractures

**DOI:** 10.1186/s13018-017-0707-8

**Published:** 2018-01-16

**Authors:** Fancheng Chen, Xiaowei Huang, Yingsun Ya, Fenfen Ma, Zhi Qian, Jifei Shi, Shuolei Guo, Baoqing Yu

**Affiliations:** 1 Shanghai Medical College, Fudan University, Shanghai City, China; 2 Department of Orthopedics, The First Affiliated Hospital of Soochow University, Suzhou, China; 3 Department of Pharmacy, Shanghai Pudong Hospital, Fudan University Pudong Medical Center, Shanghai City, China; 4 Department of Orthopedics, Shanghai Pudong Hospital, Fudan University Pudong Medical Center, No.2800 gongwei road, Huinan town, Pudong new area, Shanghai City, China

**Keywords:** Finite element analysis, Extra-articular proximal tibial fracture, Intramedullary nail, Locking plate, Comparison

## Abstract

**Background:**

Proximal tibia fractures are one of the most familiar fractures. Surgical approaches are usually needed for anatomical reduction. However, no single treatment method has been widely established as the standard care. Our present study aims to compare the stress and stability of intramedullary nails (IMN) fixation and double locking plate (DLP) fixation in the treatment of extra-articular proximal tibial fractures.

**Methods:**

A three-dimensional (3D) finite element model of the extra-articular proximal tibial fracture, whose 2-cm bone gap began 7 cm from the tibial plateau articular surface, was created fixed by different fixation implants. The axial compressive load on an adult knee during single-limb stance was imitated by an axial force of 2500 N with a distribution of 60% to the medial compartment, while the distal end was fixed effectively. The equivalent von Mises stress and displacement of the model was used as the output measures for analysis.

**Results:**

The maximal equivalent von Mises stress value of the system in the IMN model was 293.23 MPa, which was higher comparing against that in the DLP fixation model (147.04 MPa). And the mean stress of the model in the IMN model (9.25 MPa) was higher than that of the DLP fixation system in terms of equivalent von Mises stress (EVMS) (*P* < 0.0001). The maximal value of displacement (sum) in the IMN system was 8.82 mm, which was lower than that in the DLP fixation system (9.48 mm).

**Conclusions:**

This study demonstrated that the stability provided by the locking plate fixation system was superior to the intramedullary nails fixation system and served as an alternative fixation for the extra-articular proximal tibial fractures of young patients.

## Background

Proximal tibia fractures, which account for approximately 5–11% of all fractures of the tibia [1], are one of the most familiar fractures among all the long bone fractures. To maintain anatomical reduction and prevent the devastating complications, surgical approaches are usually needed [2]. However, the anatomy and surrounding soft tissues of the proximal tibia presents several exclusive treatment challenges for these injuries, and currently no single treatment method has been widely established as the standard care.

For maintaining the stability of the fractured tibial shaft, multifarious approaches have been developed. Intramedullary nailing and locking plate osteosynthesis are the two most frequently used surgical methods of treatment for extra-articular proximal tibia fractures [3, 4]. Each of these options has its own advantages and relevant complications [2]. The former has lower rates of infection and less soft-tissue dissection when compared with plates, but there is a trend towards increasing possibility of malunion with the use of nails [5]. So far, the comparative biomechanics analysis, however, has not been demonstrated computationally between these two different methods.

As an effective and accurate computational means, finite element analysis (FEA) has received extensive acceptance in the field of orthopedic research [6, 7]. The deeper insight into the stability and functionality of bone constructs can be furnished by the biomechanical studies which use the computational simulation [8, 9]. Therefore, FEA is used in this study to compare the stability of the two surgical approaches aforementioned to treat the extra-articular proximal tibial fractures. This study hypothesizes that more stress concentration existed in the intramedullary nailing osteosynthesis system, which potentially means that the intramedullary nailing is more suitable for the treatment of extra-articular proximal tibia fractures. Also, the stiffness and displacement of the constructs are evaluated to compare the fixation stability between the implants with these two methods.

## Methods

A three-dimensional (3D) geometry of an intact right lower limb, which was shared from the research our group presided over before, was generated from the CT scan of a 42-year-old Chinese healthy male with a weight of 73 kg and a height of 172 cm who has excluded comorbidities such as osteoarthritis, osteoporosis, and lower-extremity fractures.

### Experimental model

**Fig. 1:**
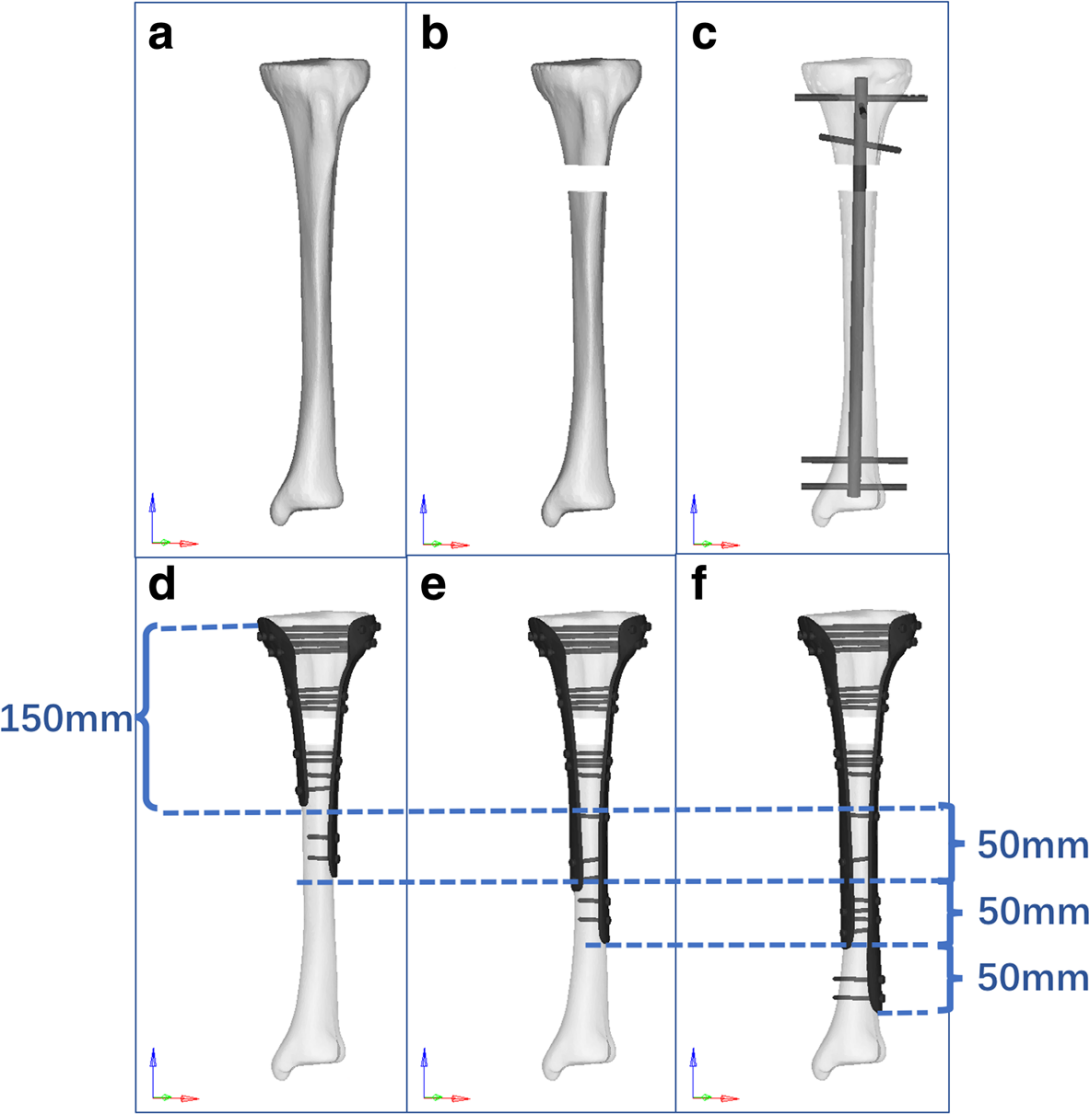
The creation of fracture model and fixation implements used in this study. **a** Intact tibia. **b** Tibia with fracture gap. **c** Model D (fixed by intramedullary nail (IN) fixation). **d** Model A (fixed by 150 mm/200 mm DLP fixation). **e** Model B (fixed by 200 mm/250 mm DLP fixation). **f** Model C (fixed by 250 mm/300 mm DLP fixation)

The serial initial CT images of the tibia and fibula were acquired with 1-mm cuts from the selected male volunteer. The CT data in the Digital Imaging and Communications in Medicine (DICOM) format were imported into the software Mimics 15.0 (Materialize Company, Leuven, Belgium) to reconstruct three-dimensional (3D) models of the tibia and fibula. The performance of further polishing and the establishment of fracture line were done by the Geomagic Studio Software (3D system Inc., Rock Hill, SC, USA). A 2-cm-transverse gap, which was 7 cm distance from the medial tibial plateau (Fig. 1b), was created to simulate a worst-case scenario of extra-articular comminuted proximal tibia fracture (OTA type 41-A3.3). The 3D models of intramedullary nails, plate, and screws were drawn by the software Creo 3.0 (Parametric Technology Corporation, USA) according to the manufacturers’ specifications.

There were four fixation models, including model A, model B, model C, and model D, set in this research to mimic the intramedullary nail fixation and different length double locking plate fixation. The models A, B, and C were fixed by double plates with a length of 150 mm/200 mm, 200 mm/250 mm, and 250 mm/300 mm, respectively. And the thickness of the plates was set as 30 mm. The proximal part of each plate was screwed by five parallel screws with a 3.5-mm diameter. Moreover, two parallel screws were located at each fracture end and the end of the plate. And there are one and three parallel screws at the middle of the distal part of the plate in models B and C, respectively. The model D was screwed by a 320-mm-length nail with three proximal screws and two distal screws. The two most proximal screws were perpendicular to each other and the bisector of positioning the third proximal screw. And the two distal screws were paralleled to the line of the lateral and medial malleolus. The diameter of the nail and screws were 10 and 4 mm, respectively. All of the screws were penetrated to the contralateral cortex as shown in Fig. 1. We employed the most common principle of screw and plate fixation based the experience of the surgeon in this study. After being positioned as recommended by the manufacturers, the models were put into the ANSYS software for re-meshing and a four-node tetrahedral three-dimensional element in this study was utilized in the selection of the unit type for the better appropriateness of geometric non-linear analysis. Table 1 shows the number of elements and nodes in each model.

**Table 1:** The number of nodes and elements in each model

Variable	Number of nodes	Number of elements
Model A	58,340	306,698
Model B	60,988	321,289
Model C	65,532	343,105
Model D	30,881	168,298

### Material properties

In this study, the bones of the tibia and fibula were defined as linear elastic material properties with Young’s modulus (E) of 17 GPa for cortical bone and 5 GPa for cancellous bone. Poisson’s ratio (y) for both cortical and cancellous bones was set at a value of 0.33 [10]. A Young’s modulus of 110 GPa and Poisson’s ratio of 0.3 were set for the properties of titanium alloy which were assigned to the simulated implant models. All the materials, including the bones and metal, were simplified as homogeneous and linear isotropic.

### Loading and boundary conditions

**Fig. 2:**
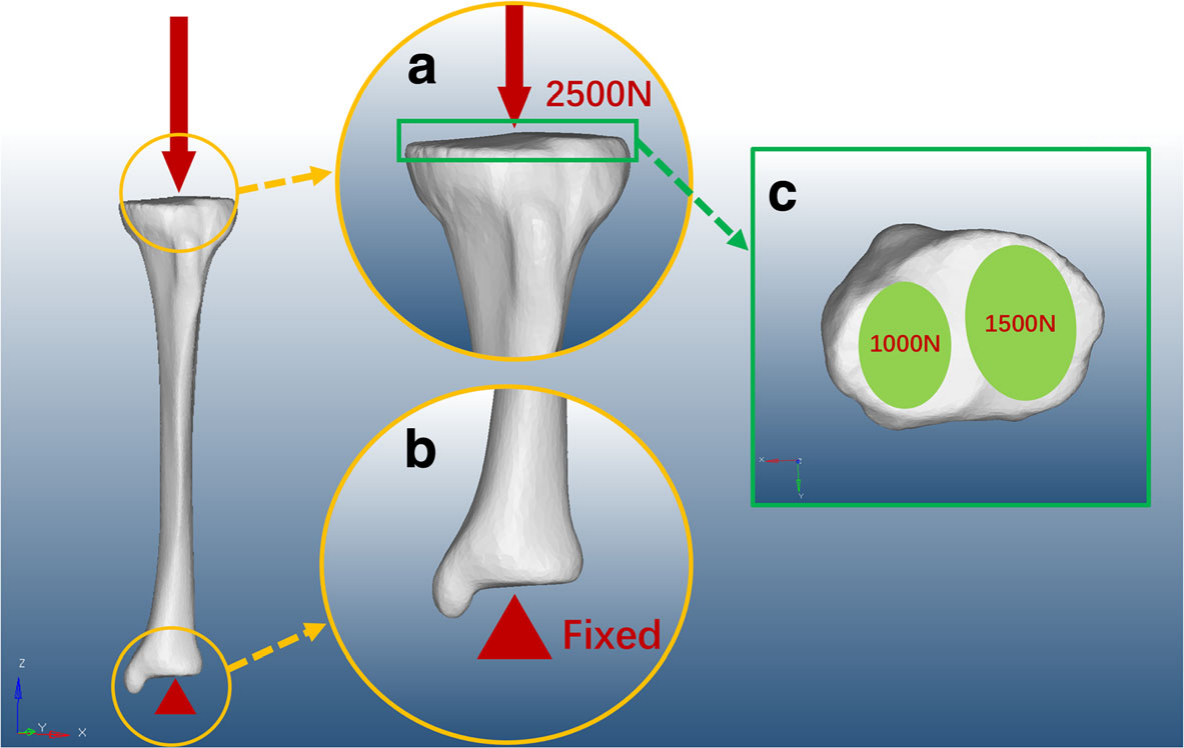
The loading and boundary conditions as well as the distribution. **a** The 2500-N vertical force established to simulate the physiological compressive load on an adult knee. **b** The distal tibia was fixed effectively. **c** The distribution of vertical force on the articular surface

A vertical force of 2500 N with a distribution of 60% to the medial tibial plateau was established to simulate the physiological compressive load on an adult knee during single-limb stance based upon the experimental data reported [11], and the distal end of the tibia was fixed effectively at the same time as shown in Fig. 2. For the interfacial surface between both the intramedullary nail and plate fixation systems, it was assumed that the direct contact with a frictional coefficient of 0.3 existed in the implants and bones [12, 13]. To imitate strong contact attachment between the plate and screw, it is defined that the interaction is sharing the common nodes of elements to simulate the locking screw mechanism.

### Analysis

In this study, the computational analysis was done using a commercial finite element software (ANSYS WORKBENCH, ANSYS. Software Corporation, Canonsburg, USA) with the equivalent von Mises stress (EVMS), displacement of the model and implants relative to the proximal tibia, which was used as the output measures. For statistical analysis, the mean values of stress and displacement between the two models were compared using Student’s *t* test. A *P* < 0.01 was regarded as statistically significant difference.

## Results

### Stress on two implants

**Fig. 3:**
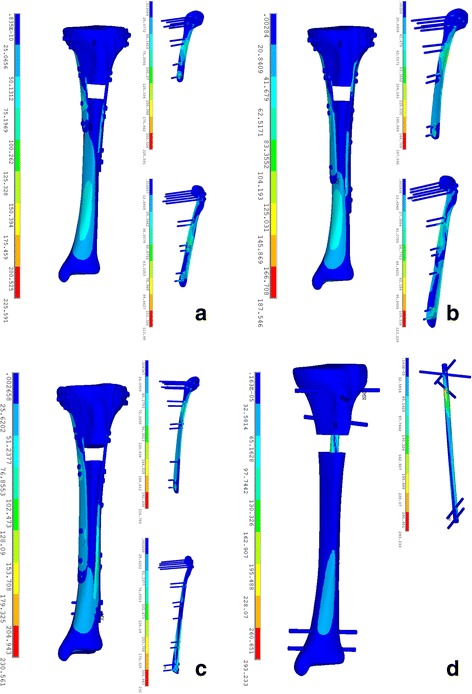
Stress distribution on models fixed with different fixation models. **a** Model A (fixed by 150 mm/200 mm DLP fixation). **b** Model B (fixed by 200 mm/250 mm DLP fixation). **c** Model C (fixed by 250 mm/300 mm DLP fixation). **d** Model D (fixed by intramedullary nail (IN) fixation)

**Fig. 4:**
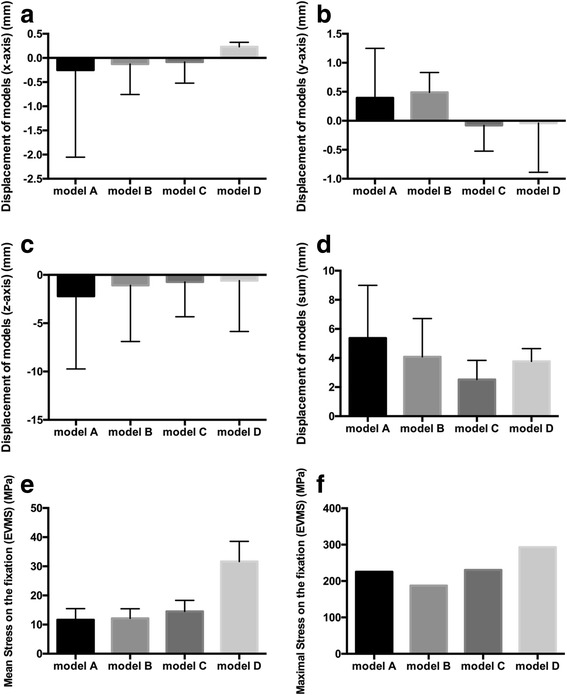
Comparison of mean stress value of models. **a** Displacement of models (*x*-axis) (mm). **b** Displacement of models (*y*-axis) (mm). **c** Displacement of models (*z*-axis) (mm). **d** Displacement of models (sum) (mm). **e** Stress on the models (EVMS) (MPa). **f** Maximal stress on the models (EVMS) (MPa)

The stress distribution in the intramedullary nail (IMN) system was different from that in the locking plate (DLP) fixation system. The maximal stress value in the models A, B, C, and D was 255.59, 187.55, 230.56, and 293.23 MPa, respectively (Fig. 3). In the IMN system, maximal stress concentrated on the intersection area between the third proximal transverse screw and the nail, as shown in Fig. 4. In the DLP fixation system, however, there was a similar characteristic that the stress was focused on the region between the fracture gap. Moreover, stress concentration was observed in the intersection area between the most distal transverse screw and the plate in the DLP system as well. The mean stress value, which was calculated from the stress of all of the nodes within the plate, was also compared between the two fixation systems. The highest mean stress of model was model D (26.80 MPa) and followed by model C which was 11.87 MPa. The third highest stress shown is in model B which is 9.8 MPa, and the lowest mean stress was 8.92 MPa in model A as listed in Table 2.

**Table 2:** The comparison of the structural results in the finite element analysis of each model

Variable	Model A	Model B	Model C	Model D
Displacement of models (*x*-axis) (mm)	− 1.53 ± 1.03***	− 0.57 ± 0.32***	− 0.39 ± 0.22***	0.16 ± 0.30
Displacement of models (*y*-axis) (mm)	− 0.21 ± 1.00***	0.25 ± 0.73***	− 0.39 ± 0.23***	− 0.64 ± 0.56
Displacement of models (*z*-axis) (mm)	− 7.52 ± 3.13***	− 5.19 ± 3.03***	− 3.28 ± 1.81***	− 4.31 ± 3.12
Displacement of models (sum) (mm)	7.93 ± 2.80***	5.94 ± 2.22***	3.45 ± 1.59***	4.39 ± 3.14

***P < 0.0001, compared with model D

### Displacement of two models

**Fig. 5:**
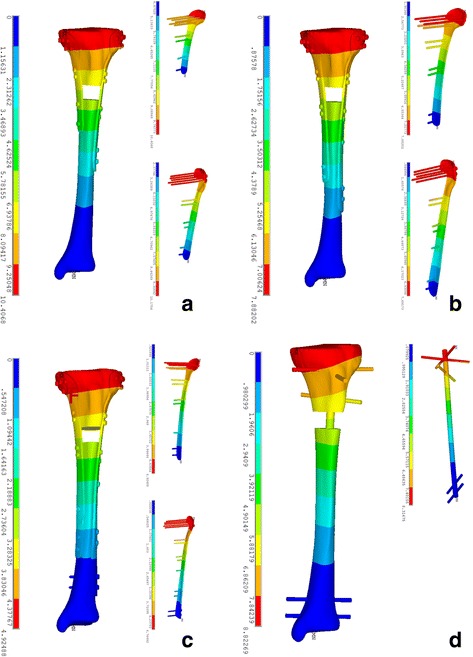
Stress distribution on models fixed with different fixation models. **a** Model A (fixed by 150 mm/200 mm DLP fixation). **b** Model B (fixed by 200 mm/250 mm DLP fixation). **c** Model C (fixed by 250 mm/300 mm DLP fixation). **d** Model D (fixed by intramedullary nail (IN) fixation)

The maximal and minimal amounts of displacement was 10.41 mm observed in model A which was fixed by 150 mm/200 mm length double locking plate and 4.92 mm in model C which was fixed by 250 mm/300 mm length plate, respectively, as shown in Fig. 5. The mean displacement (sum) of models A, B, C, and D was 7.93, 5.94, 3.45, and 4.39 mm, respectively. Moreover, there were statistically significant differences in the mean displacement between the two models in the *x*-axis, *y*-axis, and *z*-axis, respectively, (*P* < 0.001), as shown in Table 2 and Fig. 4. In addition, the mean value of displacement (sum) was also observed in model C which was 3.45 mm, which was lower than the other three models. Both of the four models manifested a similar tendency of the displacement decreasing as it approached distally.

## Discussion

Extra-articular proximal tibial fractures, which are relatively uncommon compared with tibia plateau fractures, account for approximately 5–11% of all tibial fractures [3]. Most of these fractures, which are often caused by high-energy injury and are usually associated with complicated comminution with displaced fractured fragments, need surgical approaches to maintain anatomical reduction and for preventing the development of devastating complications [14]. Intramedullary nails (IMN) and locked plating are the two most frequently used surgical methods. However, the use of the former one has been associated with malunion and is more likely to cause the loss of proximal fragment fixation [15], and several patients complained about knee pain after intramedullary nail fixation surgery [16]. On the other side, the incisions of locking plate fixation methods are longer versus the intramedullary nail, and there is more possibility of infection because of the additional injury to the soft tissue [14, 17]. Moreover, the biomechanism between these two methods had been researched and shown no difference fatigue performance between them, but the stress distribution still needs to be further studied.

Finite element(FE) analysis, in the current study, has been used for the purpose of predicting the influence of specific factors in a given system, with a view to achieving a better understanding of geometrical effects [18], because FE models can effectively focus on a single factor, negate the effects of other variables meanwhile, while the clinical studies may be influenced by several controlled and uncontrolled variables. As a result, we used the FE analysis software in this study to estimate two different fixations for treating the extra-articular proximal tibial fracture based on the 3D finite element model which mimics the extra-articular proximal tibial fracture. And we tried to explore the biomechanism distribution of these two methods.

The distribution of the stress on models was counted through equivalent Von mises stress (EVMS). The concentration of stress found on the IMN system was located on the intersection area between the third proximal transverse screw which was near the fracture gap and the nail, manifesting this screw shared an important contribution for supporting the load transmitted from the articular surface. Besides, the mean stress, as shown in Table 2, on model D was significantly higher than that on the other models. This can be explained by the fact that the double plate fixation had a bigger cross-sectional area, so the bilateral plate provided a more stable support than intramedullary nail which can endure the early weight bearing [6]. Moreover, the stress concentration was found on the cortical regions surrounding the screw holes. A similar concentration of stress was also observed on the locking plate system used in the humerus fracture. It can be understanded by the anti-sliding effect of the transverse screws, so the screws and surrounding area shared amounts of stress of whole loading. Although loading transmission through the DLP system may provide better stability, the injury of the periosteum and large incision of the surgery may cause the delayed unions especially in elderly people. As a result, the DLP fixation probably is a suitable method for young patients whose bones are biomechanically sturdy and has the requirement of early weight bearing.

In the viewpoint of biomechanics, the structures of DLP and IMN primarily supply with support in lateral and axial directions respectively. For construct stability, the IMN fixation provided the higher stiffness, which was similar to that of Lee et al. [19] who found that intramedullary nail provided a stronger stress compared with the double plates fixation. And Lee also mentioned that IMN was an ideal load sharing implant with respect to the concepts of minimally invasive surgery. However, it is necessary to note that after comparing 56 cases which included 22 intramedullary nails and 34 locking plating, Lindvall E et al. [4] pointed that IMN had a higher incidence of mal-reduction resulting in nonunion compared with locked plating. This can be explained by the ends of the fracture gap may exist micromotion because of the only loading path provided by the intramedullary nail. Therefore, mal-reduction after IMN should not be overlooked clinically, although several technical tips may help to reduce the occurrence of this complication. For example, J. Franke et al. proposed the suprapatellar nailing of tibial fractures–indications technique, which is generally performed through an infrapatellar approach, can decrease the occurrence of malalignment during intramedullary nailing of extra-articular proximal tibial fractures [20]. Moreover, the accompanying stress shielding effect can cause serious anterior knee pain and can increase the nonunion rates. However, without the normal data that determines the occurrence threshold of union rates, these concerns may be unwarranted.

On the other hand, we can find the tendency that the mean stress and displacement decreased with the increase of the length of plate. This can be explained by the longer the plate, the more area of the cortex share the load from the joint. But we also had to mention as has been said above that the longer plate caused more damage of the soft tissue. However, it is interesting to note that the maximal von Mises stress in model B was lower than the maximal stress in model C which was fixed by the longer plate, which indicated that the longer plate does not always have the lower stress focusing. It may be associated with the difference of elastic modulus between the bone and plate. But the mechanism need further study to explain. And the longer DLP fixation system also had its values. As we can observe, the stress was well-distributed on both implants and bones. In brief, in consideration of the characteristic of the DLP system, which distributes the stress more evenly and does not interrupt the loading transmission via the proximal tibial shaft, the DLP fixation system may be more suitable for the treatment in young patients whose bones are biomechanically sturdy and without osteoporosis.

It is undeniable that this study has some inherent limitations. Firstly, the materials of the cortical and cancellous bone were both simulated and probably not reflected the actual conditions, which was orthotropic, of bone properties. Secondly, soft tissues and other neighboring structures were eliminated in this study to get more direct results of comparison. Thirdly, static load was employed in this study rather than cyclic load, which may not sufficiently supersede complicated loading that may occur in activities [1], because the dynamic imitation would require considerable computer resources and time.

## Conclusion

Different load transmission mechanisms were demonstrated in this study between the locked plating fixation system and intramedullary nails fixation system. Based on these results, we believe that the double locking plate system was more stable and superior for the treatment of comminuted extra-articular fractures of the proximal tibia compared with intramedullary nail fixation from the biomechanical point of view.

## Abbreviations

DICOM: Digital imaging and communications in medicine
DLP: Double locking plate
EVMS: Equivalent von Mises stress
FEA: Finite element analysis
IMN: Intramedullary nail
